# Risk factors for oesophageal squamous cell carcinoma in Mozambique

**DOI:** 10.3332/ecancer.2022.1437

**Published:** 2022-08-04

**Authors:** Lina Cunha, Filipa Fontes*, Jotamo Come, Vitória Lobo, Lúcio Lara Santos, Nuno Lunet, Carla Carrilho

**Affiliations:** * These authors contributed equally to the manuscript; 1 Serviço de Gastroenterologia, Hospital Privado de Maputo, Rua do Rio Inhamiara, 1100 Maputo, Moçambique; 2 EPIUnit – Instituto de Saúde Pública, Universidade do Porto, Rua das Taipas, n° 135, 4050-600 Porto, Portugal; 3 Laboratório para a Investigação Integrativa e Translacional em Saúde Populacional (ITR), Universidade do Porto, Rua das Taipas, n° 135, 4050-600 Porto, Portugal; 4 Departamento de Ciências da Saúde Pública e Forenses e Educação Médica, Faculdade de Medicina da Universidade do Porto, Rua Doutor Plácido da Costa, 4200-450 Porto, Portugal; 5 Unidade de Investigação em Enfermagem Oncológica, Centro de Investigação do Instituto Português de Oncologia do Porto, Rua Dr António Bernardino de Almeida, 4200-072 Porto, Portugal; 6 Departamento de Cirurgia, Hospital Central de Maputo, 1653 Avenida Eduardo Mondlane, Maputo, Moçambique; 7 Departamento de Gastroenterologia, Hospital Central de Maputo, 1653 Avenida Eduardo Mondlane, Maputo, Moçambique; 8 Departamento de Medicina, Faculdade de Medicina, Universidade Eduardo Mondlane, 3453 Avenida Julius Nyerere, Maputo, Moçambique; 9 Departamento de Oncologia Cirúrgica, Instituto Português de Oncologia do Porto, Rua Dr António Bernardino de Almeida, 4200-072 Porto, Portugal; 10 Grupo de Investigação de Patologia e Terapêutica Experimental, Centro de Investigação do Instituto Português de Oncologia do Porto, Rua Dr António Bernardino de Almeida 4200-072 Porto, Portugal; 11 Departamento de Patologia, Faculdade de Medicina, Universidade Eduardo Mondlane, 3453 Avenida Julius Nyerere, Maputo, Moçambique; 12 Departamento de Patologia, Hospital Central de Maputo, 1653 Avenida Eduardo Mondlane, Maputo, Moçambique

**Keywords:** alcohol, diet, tobacco, oesophageal cancer, Mozambique

## Abstract

Studies evaluating risk factors for the occurrence of oesophageal squamous cell carcinoma (ESCC) in high-risk regions might contribute to a better understanding of the oesophageal cancer aetiology and incidence variation worldwide. We aimed to quantify the association between alcohol, tobacco and dietary history, and the occurrence of ESCC in Mozambique. A case–control study was conducted at Maputo Central Hospital. Cases (*n* = 143) were patients with newly diagnosed oesophageal cancer recruited in the Gastroenterology Service. Controls (*n* = 212) were selected in the Orthopaedic Ward among subjects with pathologies related to trauma. Crude and adjusted odds ratios (ORs), and the corresponding 95% confidence intervals (CI) were computed using non-conditional logistic regression. The risk of ESCC was higher in older participants and lower in those with higher household income. Alcohol drinking (lifetime consumption ≥ 55.1 versus 0 kg ethanol: OR = 5.56; 95% CI: 2.43–12.73) and tobacco smoking (lifetime consumption ≥ 20 versus 0 pack/years: OR=7.26; 95% CI: 1.42–37.17) were associated with increased risk of ESCC. Tea (at least twice daily versus less than daily: OR = 5.09; 95% CI: 2.45–10.58) was also associated with the occurrence of ESCC. No significant differences were observed for fruit and vegetable and for smoked meat or fish consumption. Findings from this study show that in our sample, the occurrence of ESCC is strongly influenced by lifetime consumption of tobacco and alcohol, and with tea drinking. This highlights the importance of preventive measures based on the promotion of healthier lifestyles.

## Introduction

According to the most recent estimates, worldwide there were more than 600,000 new cancer cases (3% of all cancers) and around 550,000 deaths due to oesophageal cancer (6% of all cancer deaths) in 2020; the highest figures for both age-standardised incidence and mortality rates are observed in Eastern Asia, and in Eastern and Southern Africa [1]. Although oesophageal cancer has two predominant histopathological subtypes, oesophageal squamous cell carcinoma (ESCC) and oesophageal adenocarcinoma (EA), ESCC has been estimated to account for approximately 90% of all oesophageal cancer cases in many sub-Saharan African countries [2–5].

In Western countries, a large number of research has been dedicated to study the determinants of ESCC [6]. In this context, tobacco smoking has been consistently considered the major risk factor for ESCC [6]; together with alcohol drinking and low consumption of fruits and vegetables, it is estimated to account for approximately 90% of the total number of cases [7]. Research from high-risk areas is more limited [8–11], and, therefore, studies assessing the relation between plausible risk factors and the occurrence of ESCC in high risk-regions, such as Eastern and Southern Africa, might contribute to a more comprehensive explanation of the oesophageal cancer incidence variation worldwide.

According to a recent systematic review of the epidemiology and risk factors of oesophageal cancer in Africa, there are no studies assessing risk factors for ESCC in Mozambique [12], although data from the Maputo Central Hospital (MCH) registry highlights that it is the fourth most frequent tumour for both sexes [13]. Therefore, this study aimed to quantify the association between alcohol, tobacco and dietary history, and the occurrence of ESCC in Mozambique.

## Material and methods

### Study design and setting

We conducted a case–control study in MCH. The MCH is a 1,500-bed quaternary hospital and the national reference hospital for cancer in Mozambique. A total of 143 cases of oesophageal cancer and 212 controls were identified between 2006 and 2010, and included in this study.

### Participants

Cases and controls were recruited among those aged 18 years or older and with sufficiently good physical and mental health to give reliable answers, as assessed by the interviewers.

Cases were patients recruited in the Gastroenterology Service of the MCH, among those with newly diagnosed oesophageal cancer. Those with histological confirmation of the diagnosis were considered eligible. From the initial number of cases included (*N* = 145), the pathological diagnosis was ESCC in 143 (98.6%) and EA in 2 (1.4%) cases; the latter were excluded from the present analysis because they were in a very small number.

Controls were selected from the Orthopaedic Ward of the MCH and included subjects with several pathologies related to trauma. Those without a diagnosis of tobacco or alcohol related diseases, or oesophagus or stomach diseases recorded as part of the admission cause, were considered eligible.

### Exposures assessment

Face-to-face interviews were conducted by trained interviewers, using a standardised questionnaire covering information on sociodemographic characteristics, coffee and tea drinking, smoking and alcohol consumption and dietary history.

Alcohol consumption was assessed through questions exploring the usual intake for each type of beverage separately, i.e. beer, wine, spirit drinks and traditional drinks (e.g. brandy, *sura*, *mahéu, canho* and* caju*). Current and ex-drinkers were asked to report the age when they started to drink and the usual number of units consumed per day, week or month, as applicable. The four types of alcoholic beverages were combined to provide an overall estimate of alcohol consumption. Ex-drinkers, defined as those who had permanently quit drinking before the interview, were asked to report at what age they stopped to drink. We estimated the lifetime cumulative quantity of alcohol consumed in kg of ethanol for each participant; for analysis, data was further categorised using the quartiles of the distribution among controls as cut-offs (0, >0 and <6.5, ≥6.5 and <23.9, ≥23.9 and <55.1 and ≥55.1 kg of ethanol). Current drinkers were dichotomised using as threshold the maximum daily limit of ethanol usually recommended for females and males (>12 g/day and >24 g/day, respectively) [14]. In addition, current drinkers of each type of beverage were categorised according to the number of drinks consumed per day.

Participants were asked if they currently were smokers of any tobacco product, and current and ex-smokers were asked to report the age when they started to smoke and the number of units consumed per day, week or month, as applicable. In addition, ex-smokers, defined as those who had permanently quit smoking before the interview, were asked to report at what age they stopped to smoke. We estimated the lifetime cumulative quantity of tobacco smoked in pack/years (average number of 20-cigarette packs per day multiplied by the number of years smoking) for each participant; for analysis data was further categorised as 0, >0 and <15, ≥15 and <20 and ≥20 pack/years.

Tea and coffee consumption were assessed by asking participants if they currently were drinkers of tea or coffee, and consumers were asked to report the number of times they drink per day, week or month, as applicable, and the usual tea or coffee temperature (very hot, hot or warm). As only one participant reported the consumption of warm tea and no one reported the consumption of warm coffee, for data analysis the ‘warm’ and ‘hot’ categories were merged.

The consumption of fruits and vegetables was evaluated through general questions on the usual intake of fruits and vegetables, and the number of times they consumed each type (fruits and vegetables) per day, week or month, as applicable; no specific questions were asked about the type of fruits or vegetables consumed. For data analysis, fruits and vegetables consumption frequency was combined and participants were categorised using the quartiles of the distribution among controls as cut-offs (< 1 time per day, ≥1 and <2 times per day and ≥ 2 times per day). In addition, participants were asked if they consumed smoked meat and/or fish.

### Data analysis

Odds ratio (OR), and the corresponding 95% confidence intervals (CI), for the association between sociodemographic characteristics, alcohol, tobacco, tea and coffee consumption and dietary history, and the occurrence of ESCC, were computed using non-conditional logistic regression, adjusted for potential confounders. Confounders were selected among those known to be associated with both the exposure and the outcome, but influenced by neither, according to previous studies and/or expert knowledge. Variables included in each model are described as footnotes of the respective table.

The potential interaction between smoking and alcohol consumption was assessed by including interaction terms in the regression models. For the purpose of this analysis, both ex-drinkers and current drinkers, and ex-smokers and current smokers, respectively, were combined in the same categories.

A sample size of 143 cases and 212 control provides a statistical power of 80%, to detect ORs ≥ 2 for exposures with 30% prevalence, at 5% significance level. Statistical analysis was conducted using STATA®, version 11.2 (StataCorp, College Station, TX, USA).

### Ethical considerations

The study protocol was approved by the Mozambican National Bioethics Committee for Health. All participants provided written informed consent.

## Results

The association between sociodemographic characteristics and ESCC is summarised in Table 1. In the multivariate analysis, the risk of ESCC was higher in older participants (>65 versus <45 years: OR = 2.39; 95% CI: 1.15–4.95) and lower in those with higher household income (>1000 versus <500 MZN per capita: OR = 0.25; 95% CI: 0.13–0.48). Although not statistically significant, a protective independent effect was observed for male participants, for those with a secondary or higher education and for those with freezer at home.

Table 2 presents the association between alcohol and tobacco consumption and ESCC. Concerning alcohol consumption, in the most complete model, ex-drinkers and current drinkers of more than 12 or 24 g of alcohol per day experienced, respectively, a 2.7- and 6.8-fold increase of risk when compared to never drinkers. When the lifetime consumption of alcohol was considered, a significant increase of risk was observed for those with higher levels of consumption (≥55.1 versus 0 kg ethanol: OR = 5.56; 95% CI: 2.43–12.73; *p*-value for trend < 0.001). In relation to the type of beverage consumed, beer was the most consumed (30.4%), followed by wine (27.3%), traditional drinks (19.7%) and spirit drinks (11.6%). Those who consumed at least one drink per day of beer and wine had an 8.5- and 6.8-fold higher ESCC risk, respectively, when compared to non-current drinkers. The strength of the association remains similar after additional adjustment to the intake of other types of beverages.

Concerning smoking, no significant differences were observed when comparing current, ex-smokers or ever smokers with never smokers but those with the highest lifetime consumption presented a significant higher risk when compared to those who never smoked (OR = 7.26; 95% CI: 1.42–37.17; *p*-value for trend = 0.197).

No significant interaction was observed between smoking and alcohol drinking (*p*-value for interaction = 0.213), despite the association between alcohol consumption and the occurrence of ESCC was lower among never smokers (OR = 1.55; 95% CI: 0.89–2.71) than among ever smokers (OR = 4.01; 95% CI: 0.78–20.57).

Table 3 shows the association between dietary habits and ESCC. There is a tendency for a higher risk with the increase of the daily frequency of consumption of tea (at least once daily versus less than daily: OR = 4.25; 95% CI: 2.16–8.35; at least twice daily versus less than daily: OR = 5.09; 95% CI: 2.45–10.58; *p*-value for trend < 0.001). No significant differences were observed across different temperatures of tea or coffee consumption, frequencies of fruit and vegetable intake and smoked meat or fish consumption.

## Discussion

To the authors’ knowledge, this is the first study evaluating risk factors for oesophageal cancer in Mozambique. We found that alcohol and tea are associated with a higher ESCC risk, while no significant association was observed for fruit and vegetable intake, smoked meat or fish consumption and coffee drinking. Regarding smoking, there was an increased risk for higher lifetime consumptions.

Alcohol drinking is widely accepted as a risk factor for ESCC [6]. Data from one systematic review on the effect of alcohol on different types of cancer showed that when comparing light drinkers, moderate drinkers and heavy drinkers with nondrinkers, the pooled relative risk for

ESCC was 1.3, 2.2 and 5.0, respectively [15]. Although direct comparisons are difficult due to the heterogeneous characteristics of the studies and the use of different criteria to classify participants according to different levels of consumption, our data supported a dose–response effect. In fact, the risk increased with the increase of the lifetime alcohol consumption (*p*-value for trend < 0.001) and, when comparing the lowest drinkers (≤12/24 g/day) and the highest drinkers (>12/24 g/day) with never drinkers, the ORs were 1.26 and 6.79, respectively. In the ESCCAPE (Esophageal Squamous Cell Carcinoma African Prevention Research) study, a large multicentre case–control study including participants from three Eastern African countries, participants from Tanzania and Kenya, who consumed between 350 and 699 g of ethanol per week, presented an approximately 2- and 3-fold higher risk of ESCC, respectively, than never drinkers; those who consumed at least 700 g of ethanol per week presented 6- and 4-fold higher risk, respectively. No significant differences were observed for ESCC risk, across different levels of ethanol intake, for Malawi participants [16].

In our data, the risk of ESCC increases with the quantity of drinks of wine and beer consumed per day, with ORs of 6.78 and 8.49, respectively, for the highest levels of intake (≥1 drink/day). Nevertheless, previous results suggested that the quantity of ethanol consumed was the most important factor in ESCC development rather than any individual type of beverage consumed [17].

Tobacco smoking has been consistently considered the major risk factor for ESCC in developed regions, accounting for 47%–57% of the total incidence in these settings [7, 18]. In our study, no significant differences were observed between current or ever smokers, and never smokers. This result is in accordance with findings from a recent study from high-risk areas of China [10] but opposite to the results of a study from Tanzania [19]. In the former, ever smokers presented a non-significant 1.3-fold higher risk when compared to never smokers [10]; in the latter, among those aged ≥ 45 years, ever smokers had a 2.0-fold higher significant risk [19]. The lack of association found in our study, could be justified, at least in part, by the small number of cigarettes smoked per day by each smoker and by the relatively low cumulative tobacco exposure. This is in accordance to the first World Health Organization Stepwise Approach to Chronic Disease Risk Factor Surveillance survey, conducted in 2005 in Mozambique, that found that most of the daily smokers reported to consume less than 5 cigarettes/day [20]. Despite the small number of participants in this exposure category, we found that those with the highest lifetime consumption (≥20 pack/years) presented a 7-fold higher risk when compared to never users. This association is strongest than those found in other case–control study in the Golestan Province, in the northeast of Iran, where those with a cumulative consumption of ≥20 pack/years presented 30% higher risk than never smokers, although in this study the differences were not statistically significant [11]. Our results highlight that in our sample, the cumulative consumption of tobacco was more important as risk factor for ESCC development than the tobacco consumption status.

The effect of some dietary aspects on ESCC risk has been previously addressed in other studies from Eastern Africa countries [21, 22]. Contrary to our findings, in a study from the Eastern Cape Province of South Africa, the authors found that males and females consuming green leafy vegetables 5–7 day/week had 38% and 50% reduced odds of developing ESCC, respectively, compared with those consuming ≤ 1 day/week; a similar reduction in odds was also observed with fruit consumption [21]. In contrary, in a previous study from Zambia, the authors found that the consumption of fruits and vegetables did not differ significantly between oesophageal cancer cases and controls [22], which is in accordance to our findings.

Regarding hot beverages, it has been noted an association with ESCC, that led the International Agency for Research on Cancer to recently classify very hot beverages (>65°C) as ‘probably carcinogenic’ to humans (Group 2A) based on epidemiological evidence, although it was noted insufficient evidence for coffee or tea when not consumed hot, which were therefore classified as ‘not classified as to its carcinogenicity to human’ (Group 3) [23, 24]. In our study, no significant differences in the ESCC risk were observed across different temperatures of tea or coffee consumption, while an increase of the risk was observed for more frequent consumers of tea or coffee, which is apparently inconsistent. We may hypothesise that this could be, at least in part, explained by the absence of participants reporting warm tea or coffee consumption, and by misclassification of some participants regarding temperature consumption. Recent data from a prospective cohort study from Iran, which used objectively measured tea drinking temperature, found that those drinking tea at ≥60ºC, compared to <60ºC, had a 1.4-fold higher incidence of ESCC [25]. In the same study, drinking self-perceived very hot tea was also associated with a 2.4-fold higher ESCC risk, when compared to cold or lukewarm tea [25]. In other study in Kenya, based on self-reported beverages temperature, the authors found a 1.4- and a 3.7-fold higher risk of ESCC among ‘hot’ drinkers and ‘very hot’ drinkers, compared to ‘warm’ drinkers. In our study, the finding of an increase of the ESCC risk with the consumption frequency is more likely to be related with a cumulative effect of the beverage temperature than an independent effect of tea or coffee.

Although this study provides new insights on risk factors for ESCC in Mozambique, some limitations need to be discussed. First, this case–control study was hospital-based and therefore more prone to selection bias. However, controls included patients with several pathologies related to trauma and not related to the exposures of interest, which may have minimised bias. Second, some misclassification of the exposures evaluated may have occurred, related with the absence of more specific questions (for instance, when evaluating the type of fruits or vegetables consumed) or the use of standardised and validated questionnaires. Furthermore, regarding tea and coffee temperature consumption, our exposure classification relies on self-reported perception of drinking temperature of beverages, which may vary across individuals. The use of the same interviews procedures in both cases and controls may have contributed to avoid any differential misclassification. Third, data regarding tea, coffee, fruit and vegetables, and smoked meat or fish consumption, were reported referring to the current consumption and may not represent changes in consumption over time. Finally, future studies may include information regarding other potential risk factors for the ESCC risk, which could be particularly important in this setting, including exposure to traditional solid biomass fuels, smokeless tobacco, mycotoxins, cooking habits, HIV status, Human Papillomavirus and *Helicobacter pylori* infections [6, 9, 26, 27], which were not evaluated in the present study.

## Conclusion

This case–control study shows that the occurrence of ESCC is strongly influenced by lifetime consumption of tobacco and alcohol, and with tea and coffee drinking. This highlights the importance of preventive public health measures based on the promotion of healthier lifestyles.

## Conflicts of interest

The authors declare that they have no conflicts of interest.

## Funding

None to be declared.

## Figures and Tables

**Table 1.** Association between sociodemographic characteristics and ESCC.

		Controls	Cases		OR (95% CI)	
		*n* (%)	*n* (%)		Crude	Adjusted^a^
Sex						
	Female	120 (56.6)	90 (62.9)		1 (reference)	1 (reference)
	Male	92 (43.4)	53 (37.1)		0.77 (0.50–1.19)	0.75 (0.45–1.23)
Age (years)						
	<45	55 (25.9)	26 (18.2)		1 (reference)	1 (reference)
	45–55	68 (32.1)	38 (26.6)		1.18 (0.64–2.18)	1.29 (0.66–2.50)
	55–65	51 (24.1)	39 (27.3)		1.62 (0.86–3.02)	1.84 (0.91–3.72)
	>65	38 (17.9)	40 (28.0)		2.23 (1.17–4.24)	2.39 (1.15–4.95)
Education (level completed)						
	None	53 (25.0)	41 (28.7)		1 (reference)	1 (reference)
	Primary	97 (45.8)	85 (59.4)		1.13 (0.69–1.87)	1.60 (0.90–2.83)
	Secondary or higher	62 (29.2)	17 (11.9)		0.35 (0.18–0.70)	0.87 (0.39–1.93)
Household income (MZN per capita)						
	<500	35 (16.5)	48 (33.6)		1 (reference)	1 (reference)
	500–999	84 (39.6)	68 (47.6)		0.58 (0.34–1.01)	0.57 (0.33–0.99)
	>1,000	93 (43.9)	27 (18.9)		0.21 (0.11–0.39)	0.25 (0.13–0.48)
Freezer (at home)						
	No	85 (40.1)	78 (54.5)		1 (reference)	1 (reference)
	Yes	127 (59.9)	65 (45.4)		0.55 (0.36–0.86)	0.77 (0.48–1.22)

OR, Odds ratio; 95% CI, 95% confidence interval

a Obtained from models including all variables presented in Table 1

**Table 2.** Association between alcohol and tobacco consumption and ESCC.

			Controls	Cases		OR (95% CI)			

			*n* (%)	*n* (%)		Crude	Adjusted^a^	Adjusted^b^	*p* for trend^b^
Alcohol drinking									
	Never drinker		99 (46.7)	60 (42.0)		1 (reference)	1 (reference)	1 (reference)	
	Ever drinker (ex- and current drinkers)		113 (53.3)	83 (58.9)		1.21 (0.79–1.86)	1.68 (1.02–2.76)	1.68 (0.97–2.91)	--
Alcohol drinking									
	Never drinker		99 (46.7)	60 (42.0)		1 (reference)	1 (reference)	1 (reference)	
	Ex-drinker		27 (12.7)	24 (16.8)		1.47 (0.78–2.77)	2.42 (1.16–5.04)	2.73 (1.18–6.33)	
	Drinker (≤12 g/day for females and ≤24 g/day for males)		82 (38.7)	46 (32.2)		0.93 (0.57–1.50)	1.22 (0.70–2.13)	1.26 (0.60–2.29)	
	Drinker (>12 g/day for females and >24 g/day for males)		4 (1.9)	13 (9.1)		5.36 (1.67–17.20)	6.23 (1.79–21.62)	6.79 (1.81–25.46)	0.080
Lifetime alcohol consumption (kg ethanol)									
	0		99 (46.7)	60 (42.0)		1 (reference)	1 (reference)	1 (reference)	
	>0 and <6.5		28 (13.2)	16 (11.2)		0.94 (0.47–1.89)	1.18 (0.56–2.51)	1.11 (0.51–2.46)	
	≥6.5 and <23.9		29 (13.7)	9 (6.3)		0.51 (0.23–1.16)	0.67 (0.28–1.62)	0.76 (0.30–1.94)	
	≥23.9 and <55.1		28 (13.2)	11 (7.7)		0.65 (0.30–1.40)	1.32 (0.55–3.20)	1.62 (0.61–4.27)	
	≥55.1		28 (13.2)	47 (32.9)		2.77 (1.57–4.88)	5.01 (2.41–10.42)	5.56 (2.43–12.73)	<0.001
Current type of alcohol consumption (drinks/day)									
	Beer								
		0	158 (74.5)	100 (69.9)		1 (reference)	1 (reference)	1 (reference)	
		>0 and <1	49 (23.1)	32 (22.4)		1.03 (0.62–1.72)	1.11 (0.63–1.97)	1.16 (0.64–2.13)	
		≥1	5 (2.4)	11 (7.7)		3.47 (1.17–10.30)	6.75 (1.90–24.03)	8.49 (2.00–36.16)	0.032
	Wine								
		0	147 (69.4)	100 (69.9)		1 (reference)	1 (reference)	1 (reference)	
		>0 and <1	62 (29.2)	38 (29.6)		0.90 (0.56–1.45)	1.02 (0.59–1.78)	1.04 (0.57–1.89)	
		≥1	3 (1.4)	5 (3.5)		2.45 (0.57–10.48)	5.10 (1.08–24.12)	6.78 (1.26–36.60)	0.218
	Spirits								
		0	188 (88.7)	126 (88.1)		1 (reference)	1 (reference)	1 (reference)	
		>0 and <1	23 (10.8)	16 (11.2)		1.04 (0.53–2.04)	1.42 (0.65–3.11)	1.66 (0.70–3.92)	
		≥1	1 (0.5)	1 (0.7)		1.49 (0.09–24.07)	2.53 (0.14–44.46)	0.96 (0.04–22.30)	0.321
	Traditional								
		0	168 (79.2)	117 (81.8)		1 (reference)	1 (reference)	1 (reference)	
		>0	44 (20.8)	26 (18.2)		0.85 (0.49–1.45)	0.84 (0.45–1.56)	0.85 (0.44–1.65)	0.634
Tobacco consumption									
	Never smoking		149 (70.3)	106 (74.1)		1 (reference)	1 (reference)	1 (reference)	
	Ever smoking (ex- and current smoking)		63 (29.7)	37 (25.9)		0.83 (0.51–1.33)	1.09 (0.61–1.99)	1.05 (0.54–2.01)	--
Tobacco consumption									
	Never smoking		149 (70.3)	106 (74.1)		1 (reference)	1 (reference)	1 (reference)	
	Ex-smoker^c^		41 (19.3)	18 (12.6)		0.62 (0.34–1.13)	0.83 (0.41–1.69)	0.80 (0.37–1.76)	
	Smoker^c^		22 (10.4)	19 (13.3)		1.21 (0.63–2.35)	1.62 (0.73–3.57)	1.48 (0.63–3.47)	0.519
Lifetime tobacco consumption (pack/years)									
	0		149 (70.3)	106 (74.1)		1 (reference)	1 (reference)	1 (reference)	
	>0 and <15		52 (24.5)	25 (17.5)		0.68 (0.39–1.16)	0.86 (0.45–1.63)	0.81 (0.40–1.63)	
	≥15 and <20		8 (3.8)	3 (2.1)		0.53 (0.14–2.03)	0.81 (0.16–4.01)	0.79 (0.15–4.13)	
	≥20		3 (1.4)	9 (6.3)		4.22 (1.12–15.95)	6.38 (1.48–27.40)	7.26 (1.42–37.17)	0.197

OR, Odds ratio; 95% CI, 95% confidence interval

a Obtained from models including all variables presented in Table 1

b Obtained from models including all variables in Table 1, tea consumption (less than daily, once daily, at least twice daily), coffee consumption (less than daily, at least once daily), fruits and vegetables consumption (< times per day, ≥1 and < times per day, ≥2 times per day) and smoked meat or fish consumption (no, yes). In addition, for alcohol variables, the model also included tobacco consumption (never smoking, ever smoking) and for tobacco variables, the model also included alcohol consumption (never drinker, ever drinker)

c The median (percentile 25–percentile 75) number of cigarettes consumed per day was 6 (3–15) among ex-smokers and 5 (3–10) among smokers. The median (percentile 25–percentile 75) years of tobacco consumption was 17 (9–26) among ex-smokers and 31 (25–40) among smokers

**Table 3.** Association between dietary history and ESCC.

		Controls	Cases		OR (95% CI)			*p* for trend^b^
		*n* (%)	*n* (%)		Crude	Adjusted^a^	Adjusted^b^	
Tea consumption (frequency)								
	Less than daily^c^	77 (36.3)	18 (12.6)		1 (reference)	1 (reference)	1 (reference)	
	Once daily	82 (38.7)	76 (51.8)		3.86 (2.12–7.05)	4.19 (2.20–7.99)	4.25 (2.16–8.35)	
	At least twice daily	53 (25.0)	51 (35.7)		4.12 (2.17–7.82)	5.02 (2.49–10.1)	5.09 (2.45–10.58)	<0.001
Tea consumption (temperature)^d^								
	Warm/hot	165 (79.7)	117 (81.8)		1 (reference)	1 (reference)	1 (reference)	
	Very hot	42 (20.3)	26 (18.2)		0.87 (0.51–1.50)	0.85 (0.48–1.54)	0.81 (0.44–1.49)	--
Coffee consumption (frequency)								
	Less than daily^e^	202 (95.3)	133 (93.0)		1 (reference)	1 (reference)	1 (reference)	
	At least once daily	10 (4.7)	10 (7.0)		1.52 (0.61–3.75)	3.18 (1.09–9.29)	3.15 (0.97–10.17)	--
Coffee consumption (temperature)^f^								
	Warm/hot	148 (86.6)	44 (88.0)		1 (reference)	1 (reference)	1 (reference)	
	Very hot	23 (13.4)	6 (12.0)		0.88 (0.34–2.29)	0.65 (0.23–1.85)	0.51 (0.16–1.65)	--
Fruit and vegetable consumption								
	<1 times per day	97 (46.0)	68 (48.2)		1 (reference)	1 (reference)	1 (reference)	
	≥1 and <2 times per day	62 (29.4)	44 (31.2)		1.01 (0.62–1.66)	1.24 (0.72–2.13)	1.05 (0.59–1.87)	
	≥2 times per day	52 (24.6)	29 (20.6)		0.80 (0.46–1.38)	0.93 (0.51–1.67)	0.84 (0.45–1.57)	0.639
Smoked meat or fish consumption								
	No	186 (88.2)	132 (92.3)		1 (reference)	1 (reference)	1 (reference)	
	Yes	25 (11.8)	11 (7.7)		0.62 (0.28–1.30)	0.53 (0.24–1.18)	0.54 (0.23–1.29)	--

OR, Odds ratio; 95% CI, 95% confidence interval

a Obtained from models including all variables presented in Table 1

b Obtained from models including all variables presented in Table 1, alcohol drinking (never drinking, ever drinker), tobacco consumption (never smoking, ever smoking) and tea consumption (less than daily, once daily, at least twice daily), coffee consumption (less than daily, at least once daily), fruits and vegetables consumption (< times per day, ≥1 and < times per day, ≥2 times per day) and smoked meat or fish consumption (no, yes), as applicable

c Includes four non-drinkers

d Data available for 350 tea drinkers

e Includes 134 non-drinkers

f Data available for 221 coffee drinkers
